# Mn-Promoted Co/TiO_2_ Catalysts: Quantitative Analysis of Cobalt Polymorphs and Stacking Faults and Its Effect on Fischer-Tropsch Synthesis Performance

**DOI:** 10.1021/acscatal.5c07197

**Published:** 2026-02-05

**Authors:** Danial Farooq, Lucy Costley-Wood, Sebastian Stockenhuber, Antonis Vamvakeros, Stephen W. T. Price, Lisa J. Allen, Jakub Drnec, James Paterson, Mark Peacock, Daniel J. M. Irving, Philip A. Chater, Andrew M. Beale

**Affiliations:** † Department of Chemistry, 4919 University College London, 20 Gordon Street, London WC1H 0AJ, U.K.; ‡ Rutherford Appleton Laboratories Research Complex at Harwell, Harwell Science and Innovation Campus, Harwell, Didcot OX11 0FA, U.K.; § Finden, Building R71 Harwell Campus, Oxfordshire OX11 0QX, U.K.; ∥ European Synchrotron Radiation Facility, ID 31 Beamline, BP 220, Grenoble Cedex F-38043, France; ⊥ BP Applied Sciences, Innovation & Engineering, Saltend, Hull HU12 8DS, U.K.; # 120796 Diamond Light Source Ltd, Diamond House, Harwell Campus, Didcot, Oxfordshire OX11 0DE, U.K.

**Keywords:** cobalt, Fischer-Tropsch, XRD-CT, faulting, HCP

## Abstract

The transition to net-zero emissions hinges on circular economy strategies that valorize waste and enhance resource efficiency. Among X-to-liquid (XTL) technologies, the Fischer-Tropsch (FT) process stands out for converting biomass, waste, and CO_2_ into hydrocarbons and chemicals, especially when powered by renewable hydrogen. Cobalt-based catalysts are preferred in FT synthesis due to their efficiency and CO_2_ tolerance, yet their catalytic performance is closely tied to their polymorphic structures—face-centered cubic (FCC), hexagonal close-packed (HCP), and stacking-faulted intergrowths thereof. HCP cobalt has been shown to exhibit high activity and selectivity for higher hydrocarbons and oxygenates, particularly when transformed into cobalt carbide (Co_2_C), which forms more readily at low H_2_/CO ratios. This study presents a quantitative analysis of cobalt polymorphs and stacking faults in Mn-promoted Co/TiO_2_ FT catalysts from in situ powder X-ray diffraction (XRD) data and X-ray Diffraction Computed Tomography (XRD-CT) data from spent catalysts in order to obtain a more complete correlation of structural features with catalytic performance. By modeling stacking fault probabilities using supercell simulations, the proportion of faulted FCC and HCP domains was determined across varying Mn loadings (0–5%). Increased Mn loading was found to decrease stacking faults in the FCC phase while increasing them in HCP, promoting the formation of HCP domains and ultimately Co_2_C under reaction conditions. Notably, the 3% Mn-loaded sample showed a marked rise in HCP content and Co_2_C formation, correlating with the highest observed alcohol and olefin selectivity. These findings highlight a critical structure–function relationship: Mn facilitates a transformation from FCC to HCP and then to Co_2_C, this final transition driven by similar stacking sequences and metal–support interactions. The findings show that Mn promotion not only stabilizes smaller Co particles and enhances its dispersion, but also modulates the distribution of Co polymorphs and stacking faults, leading to altered catalytic behavior. This highlights the importance of stacking fault characterization for optimizing FT catalyst design and performance, and suggests pathways to more efficient and selective carbon-neutral fuel production through engineered polymorphic and interfacial structures.

## Graphical abstract

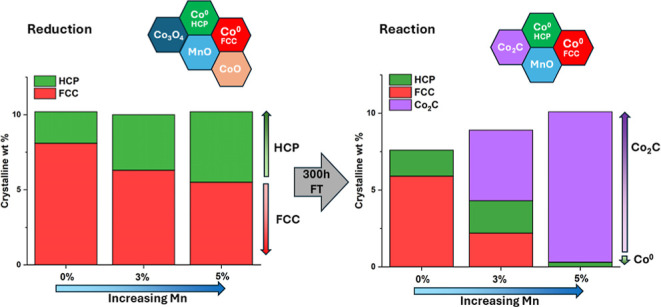

## Introduction

The transition to net-zero emissions by 2050 depends on adopting circular economy principles that promote resource efficiency and waste valorization. X-to-liquid (XTL) technologies, especially the Fischer-Tropsch (FT) process, offer promising routes to convert biomass, waste, and captured CO_2_ into valuable hydrocarbons and chemicals. Originating in the 1920s, FT involves the conversion of syngas (CO and H_2_) into liquid fuels. When powered by renewable hydrogen, it offers a path to carbon-neutral fuels and additionally can be tuned to produce high-value olefins and alcohols. Key catalysts include cobalt, iron, nickel, and ruthenium, though cobalt has become industrially preferred for its efficiency and CO_2_ tolerance.^1–4^ Notable applications include Shell’s gas-to-liquid (GTL) Pearl plant in Qatar, the current largest GTL plant containing more than 5000 miles worth of reactor tubes,^5^ and BP-Matthey’s demonstration plant in Alaska. Strategic biofuels is also deploying FT technology to transform forestry waste into renewable diesel, and a new FT sustainable aviation plant was announced by DG Fuels in 2024 which will be the largest of its kind,^6^ together underlining the FT process’s growing role in sustainable fuel and chemical production.

Metallic cobalt (Co) primarily exists in two polymorphs: face-centered cubic (FCC) and hexagonal close-packed (HCP), though research has also identified the existence of a body-centered cubic (BCC) polymorph.^7^ In FT catalysis, both FCC and HCP Co phases are recognized for their activity; however, most research indicates the superior activity of the HCP Co phase.^8–13^ This advantage is attributed to its direct CO dissociation reaction mechanism compared to the hydrogen-assisted CO dissociation route typically followed by the FCC phase.^8^ The HCP structure also transforms more readily into cobalt carbide (Co_2_C) due to a similar stacking sequence (ABAB).^9,14^ Co_2_C is stable under FT conditions and tends to form more readily at lower H_2_/CO ratios and temperatures, and despite being previously regarded as catalytically inactive, or even responsible for the deactivation of Co catalysts, recent studies have shown it plays a crucial role in enhancing alcohol selectivity.^15^ Operando XRD investigations by Van Ravenhorst et al. demonstrated the transformation of FCC cobalt to Co_2_C without significant catalyst deactivation, and suggest that any loss in activity may be compensated by a rise in the HCP phase, which they observed to occur concurrently to Co_2_C formation.^16^ Further insights have come from Zhao et al., who studied model catalysts featuring interfaces between metallic cobalt and Co_2_C phases.^17^ They found that systems with either Co on Co_2_C, or Co_2_C on Co, showed similar alcohol selectivity, indicating that the interface between the two phases is likely the active site for alcohol formation. Additionally, Co on Co_2_C promoted higher olefin selectivity, attributed to operating under lower H_2_/CO syngas ratios. Mechanistically, alkyl chain formation involves both CO dissociation and hydrogenation, whereas alcohol formation involves nondissociative CO adsorption and insertion at the Co–Co_2_C interface.^17^

Promoters such as manganese (Mn) have shown significant promise in enhancing FT catalyst performance. Mn-promoted Co catalysts display higher activity, increased C_5_^+^ hydrocarbon and olefin selectivity, and lower methane production due to a decreased H_2_ uptake, suppressing hydrogenation activity, and a reduced CO dissociation barrier.^2,18–21^ Yang et al. attribute these changes in performance to a Co_2_C rich surface, with its formation facilitated by the Mn,^22^ with Pedersen et al. agreeing that Mn promotes the disproportionation and dissociation of CO, required for Co_2_C formation, via Lewis acid–base interactions at Mn^2+^ sites on MnO clusters.^19^ This Co_2_C phase is thermodynamically stabilized on Mn-promoted Co, due to enhanced cobalt dispersion, reduced particle size, and increased surface area.

This enhanced carbide formation is not unique to Mn, and has been observed for a range of dopants, for example Na and La.^3,23^ TiO_2_ is a well-studied support material for Co catalyzed FT, considered advantageous due to its high surface area, chemical stability and propensity for strong metal–support interaction with Co.^24^ Its high porosity and pore size also enable high Co metal dispersion.^25^

While Co FT catalysts are often profiled to contain distinct FCC and HCP phases, XRD analysis regularly identifies that these catalysts comprise stacking-faulted, intergrown FCC/HCP phases.^3,26^ In general, stacking faults in FCC metals occur when the [110] Burgers vector dislocation splits into partial dislocations with a [112] Burgers vector on the same plane.^27^ In HCP metals, the type of dislocation depends on the c/a ratio of the unit cell, leading to distinct slippage modes. Both FCC and HCP phases possess similar formation energies^28^ and overlapping atomic stacking sequences, which can result from a disordered polytypic structure.^29^ Stacking faults are quantitatively measured using the stacking probability, *P*_stack_, indicating the probability of ABC stacking, corresponding to FCC and HCP structures at values of 0 and 1 respectively. Experimentally, stacking faults result in lower-than-expected intensities of the (200) peak for FCC phases, and broad and distorted peak shapes in both FCC and HCP phases. This structural broadening renders quantitative phase analysis and crystallite size calculations, very inaccurate though the effect is less prominent for the (220)_FCC_/(110)_HCP_ and (311)_FCC_/(112)_HCP_ reflections.^9,30^ Furthermore, the presence of stacking faults can create unique active sites as well as affecting electronic structure; indeed it has long been thought that the differences in Cu nanoparticles used in CO_2_ conversion can be traced to the subtle differences in Bragg reflection intensity and position caused by stacking faulting.^31,32^ Metals of catalytic interest prone to this phenomenon tend to adopt (kinetically) stable alternative polymorphic forms at the nanoscale and include Ni, Ru, Ag etc and increasingly this polymorphism is being investigated/characterized more deeply in an ultimate attempt to exploit for the design of more active species i.e. fuel cells/electrolyzers.^33–35^ For FTS catalysts, a more thorough characterization of the metal species has to date proven something of a blind spot rendering it increasingly important to better understand the structure of such intergrown species, and to determine their influence on catalytic performance.^20^

Scattering techniques can be employed to characterize stacking faults within disordered mixed FCC/HCP Co metals. Recent examples include the use of statistical correlations of atomic layers to simulate XRD patterns,^36^ the simulation and refinement of models against the diffraction patterns for different stacking fault levels using specialized software including DIFFaX^37^ and FAULTS,^38^ computational approaches involving the building of supercells,^39,40^ even the application of pair distribution function (PDF) analysis.^41,42^ Notably, the addition of X-ray diffraction computed tomography (XRD-CT) brings such studies closer to industrial application, allowing the location of such phases to be spatially resolved across a catalyst pellet, not only a model powder. Previous work utilizing this technique by Price et al. identified the increased contribution of intergrown FCC/HCP Co under operando FT conditions, however no Co_2_C was observed.^43^ More recent work by Farooq. et al, the authors of the current paper, were able to identify Co_2_C on Mn-promoted Co Ft catalysts, and observed a higher concentration of the carbide phase on the periphery of the extrudate.^18^ In both studies, however, stacking faults are observed, and known to be of influence on the reaction, but their exact role and the extent of their contribution to performance not unravelled.

Building on this work, the present study thoroughly explores the structure of FCC/HCP intergrown Co phases in Mn-promoted Co/TiO_2_ FT catalysts, in both time-and space-resolved experiments. Careful refinements of the reduced powdered catalysts are coupled with XRD-CT measurements of spent catalyst pellets “suspended in animation” after reaction under industrially relevant conditions yet preserved in the wax product, providing precise knowledge of the phase distributions before and after reaction. Importantly, quantitative stacking fault analysis outlines the effect of Mn loading on the stacking fault density and Co_2_C formation, and combined with activity and selectivity data, yields information on the specific activity of FCC/HCP intergrown phases, and its contribution to total catalyst performance.

## Methodology

### Synchrotron PXRD

Powder X-ray diffraction patterns of catalysts immediately following thermal reduction were measured on the I15-1 beamline at the diamond light source (DLS). These were Co_3_O_4_/TiO_2_ catalytic extrudates (crushed to form 20 mg of a ∼100 μm sieve fraction) with varying Mn loading (0, 3, 5, and 10 wt %), loaded into 3 mm diameter capillaries. Measurements were performed using a wavelength of 0.1896 Å (65.4 keV) and a 1 s acquisition time, with a sample-detector (Pilatus 100 K) distance of 854 mm. XRD images were calibrated using a CeO_2_ standard reference, and an empty quartz capillary was measured for reference. Samples were measured in capillaries, following reduction at 400 °C in 10 mL/min of 4% H_2_/Ar. Only measurements postreduction, where FCC/HCP Co metal was known to be present, were relevant to this study.

### XRD-CT of Catalyst Pellets

Trilobe extrudates containing 10 wt % cobalt and varying manganese loadings (0, 1, 2, 3, 5, and 10 wt %) were synthesized by dissolving cobalt nitrate hexahydrate and manganese acetate tetrahydrate in water, followed by the addition of P25 titania powder. The resulting solution was impregnated onto the titania, thoroughly mixed, and extruded into trilobe-shaped pellets with a diameter of 1.6 mm. These extrudates were dried at 120 °C for 24 h and subsequently calcined at 300 °C in a box furnace.

Reduction was carried out at 300 °C under 100% H_2_at atmospheric pressure for 25 h prior to syngas exposure. All catalysts maintained a fixed cobalt loading of 10 wt %, with increasing manganese content resulting in a corresponding decrease in TiO_2_ proportion. One sample with 10 wt % Mn underwent an additional activation step at 450 °C.

Each catalyst (1 g) was tested under FT conditions for 300 h using a syngas mixture of H_2_/CO = 2:1, at a gas hourly space velocity (GHSV) of 3000 h^–1^, 30 barg pressure, and temperatures ranging from 210 to 240 °C. Catalysts with higher Mn loadings required elevated temperatures to achieve comparable conversion rates—see Figure 1.

**Figure 1:**
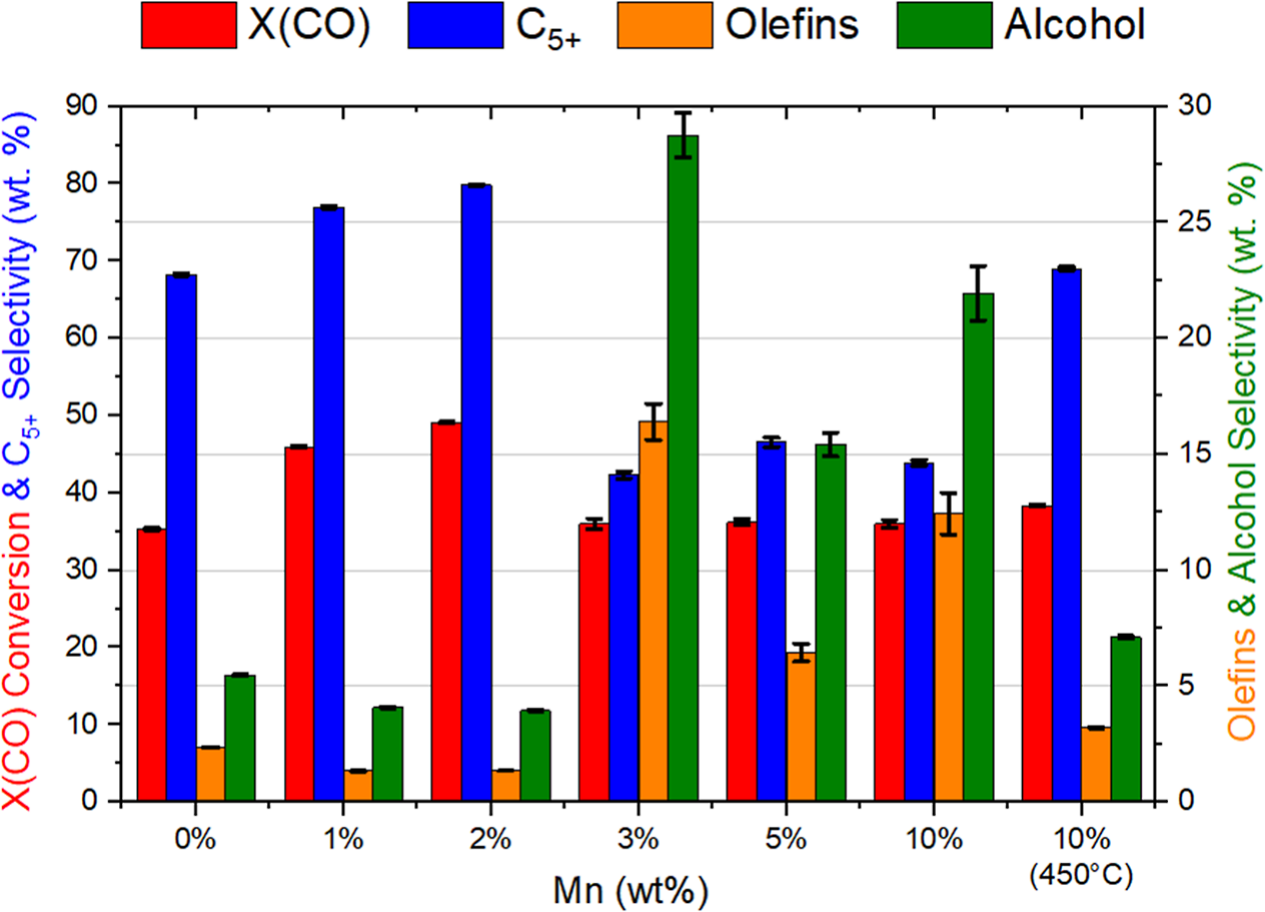
Average CO conversion and C_5+_, alcohol and olefin selectivity (150–270 h) as a function of Mn loading. Note lower CO conversion when [Mn] exceeds 3% but also significantly greater olefin and alcohol selectivity. Due to the complexity of the product distribution, it is difficult to completely close the carbon mass balance and hence typical values are reported to span 95–105%.^18^

Online gas chromatography (GC) was employed to monitor key performance metrics including CO conversion, short-chain hydrocarbon selectivity, and overall productivity, following protocols established in a previous study.^18^ Conversion and selectivity were determined by comparing the Ar internal standard with inlet and outlet CO concentrations. Hydrocarbon distributions from C_1_ to C_20_ were measured, and selectivity for C_5_+ products was calculated as 100 minus the sum of C_1_–C_4_ fractions.

Experiments were conducted in an 8-channel high-throughput reactor system, featuring shared gas feeds and pressure control, but independent temperature regulation for each channel. Catalysts were loaded into individual liners, followed by leak testing, activation, and FT synthesis. Following the reaction, the extrudates were recovered with the in situ-generated wax coating left intact. Earlier work demonstrates that this wax layer restricts oxygen diffusion to the smaller (≤7 nm) Co crystallites, which are highly sensitive to oxidation, thereby functioning as a self-passivating layer. Only a brief N_2_ purge was applied. Note the performance of these catalysts has previously been reported and discussed elsewhere.^18^

Quartz wool was used to secure the pellets in glass capillaries (3 mm diameter, 0.1 mm wall thickness) for X-ray diffraction-computed tomography (μ-XRD-CT) measurements. The μ-XRD-CT scans were conducted on beamline ID31 at the European synchrotron radiation facility (ESRF). A monochromatic pencil X-ray beam of 0.1362 Å (91 keV) with a size of 5 × 22 μm, a 50 ms acquisition time, and a PILATUS CdTe 2M detector were employed. Tomographic scans were performed using a motorized stage with 249 translation positions and 120 rotation angles over a 180° range in an interlaced approach lasting ∼5 min for one slice. The reconstructed cross sections were sampled on a 249 × 249 pixel grid.^44^ The sample-detector distance was 370 mm. XRD images were calibrated using a CeO_2_ standard reference.

Following the calibration of each 2D diffraction image, pyFAI software and Python scripts were employed for azimuthal integration, transforming the images into 1D powder diffraction patterns.^44,45^ Subsequently, air scattering was eliminated, and sinograms were centered using MATLAB scripts (MATLAB, 2020). The filtered back projection algorithm was utilized for the reconstruction of XRD-CT data, resulting in a three-dimensional array (249 × 249 × 924). In this array, the 249 × 249 pixels represented the size of the 2D cross-section image, while the 924 points stored the complete diffraction pattern for each pixel. The spatial resolution of each pixel was approximately 20 μm. The pixel patterns were summed to generate mean patterns for each sample for an initial analysis; this resultant model was then used to extract information on the crystalline phases in each pixel. However, to simplify the presentation of the data, MATLAB was employed to segment the trilobe pellets into five layers, each being three to four pixels thick, facilitating analysis based on the distance from the pellet center.

### Modeling of Stacking Faults

Ideal FCC and ideal HCP unit cells were modeled as a sequence of individual atomic layers, with transition vectors defined between the layers. This is illustrated in Figure S1 where the FCC polymorph is formed from a regular sequence of S1 vectors and the HCP phase from alternating S1 and S2 vectors. TOPAS V6 was used to model the individual layers and define stacking vectors to the subsequent layers to produce XRD patterns for the FCC and HCP phases.^46^ Similar simulations using TOPAS have been conducted.^40,47,48^ These models were refined against ideal FCC/HCP computed patterns to verify the layered structures (Figure 2).

**Figure 2:**
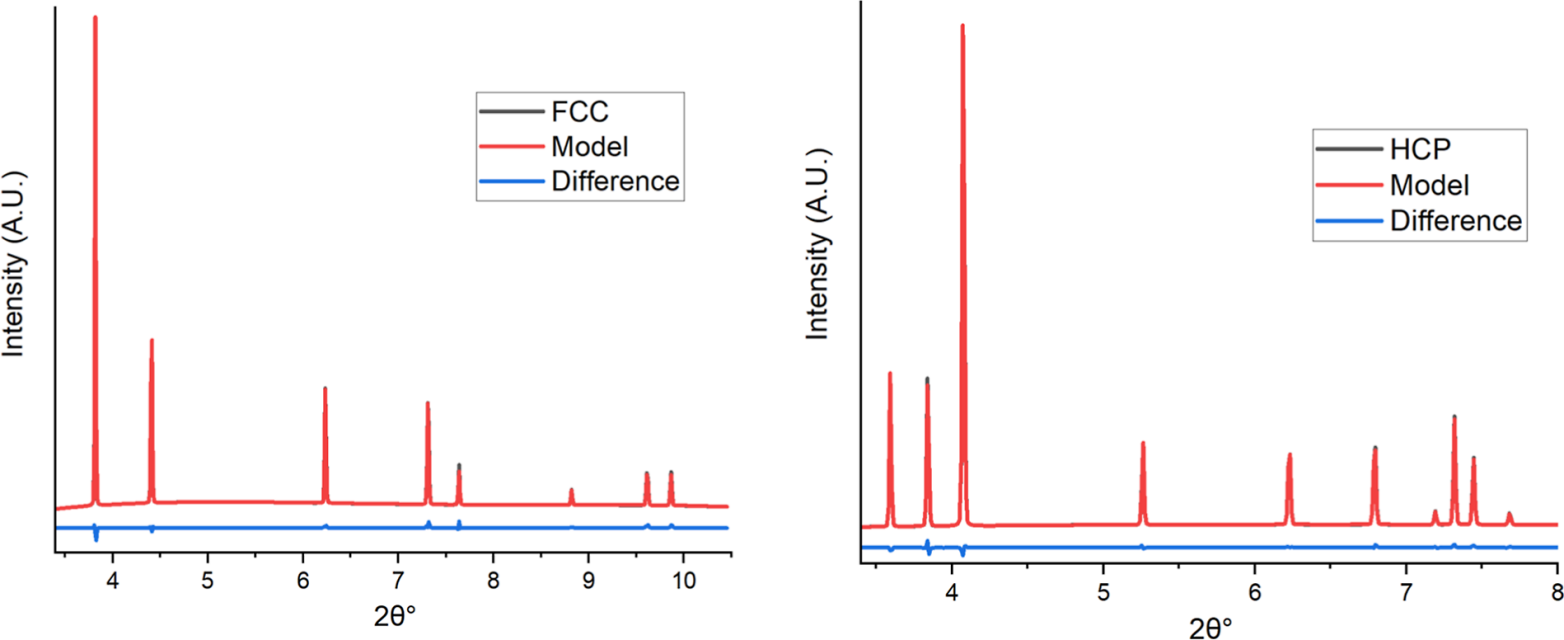
(Left) ideal FCC stacking model refined against calculated FCC structure to verify the modeled stacking structure with a *R*_wp_ = 1.6%. (Right) ideal HCP stacking model refined against calculated HCP structure to verify the modeled stacking structure with a *R*_wp_ = 2.2%. Both were calculated using a wavelength of 0.1362 Å.

Stacking faults were implemented in the FCC structure as a single S2 vector, resulting in an intrinsic fault with a probability of *P*_a_ and a subsequent S2 vector resulting in an extrinsic fault with a probability of *P*_b_. A fault in the HCP structure was identified as an S1 vector following a preceding S1 vector, with a probability *P*_c,_ as represented in Figure S2. Therefore, a *P*_a_ value of 0 corresponds to a perfect FCC structure, while a *P*_a_ value of 1 and a *P*_b_ value of 0 corresponds to a perfect HCP structure. Furthermore, a *P*_c_ value of 0 corresponds to a perfect HCP structure, while a value of 1 corresponds to an FCC structure. Both models were included to investigate the presence of FCC and HCP dominated phases. 100 sequences were generated with 100 stacks per sequence for each phase in the program; the same approach was utilized by Bette et al. explicitly note that their three-dimensional grid search always led to the same global minimum provided ≥100 supercells (with sufficiently long stacks) are averaged.^48^ TOPAS was not able to directly refine the stacking fault probabilities. As an alternative, a grid search optimization approach was adopted where refinements were executed in the program iteratively using MATLAB, systematically exploring values for *P*_a_, *P*_b_, and *P*_c_ with 0.05 increments between 0 and 1. This iterative process involved 9261 (21^3^) cycles and took ∼10^4^ s to run for each pattern. The optimal stacking fault probabilities were selected based on the lowest *R*_wp_ value, which indicated the best fit.

*P*_a_ represents the total stacking fault probability, which is the sum of intrinsic and extrinsic probability, while *P*_b_ is the extrinsic fault proportion of total fault probability (*P*_a_). This is due to an extrinsic fault being formed by a second consecutive S2 vector after an initial intrinsic fault. The resultant probabilities of intrinsic faulting (*i*) and extrinsic faulting (*e*) are defined in eqs 1.1 and 1.2, respectively, found in the Supporting Information.

## Results and Discussion

### Stacking Fault Analysis in Powdered Forms of the Catalyst Post Reduction

The XRD patterns measured at the end of the in situ reduction process were refined to quantify the presence of FCC/HCP stacking faults in the catalysts as a function of Mn loading. Note that no Mn containing phases were identified in the PXRD patterns, consistent with previous studies; following thermal reduction the Mn has been shown to be present as small MnO clusters although after reaction and with increasing [Mn], speciation is more varied.^18,21^ The refinement of the 3% Mn sample is presented in Figure 3, with and without the inclusion of stacking faulting in the models. The FCC (200) peak is diminished in the experimental data, impacting the fit of the HCP (111) peak, as the refinement program struggles to match the intensity of the HCP (111) peak while also accounting for the diminished FCC (200) peak. By including the stacking faulted model the fit notably improves, and the residual weight percentage (*R*_wp_) reduced from 7.1 to 3.4%.

**Figure 3:**
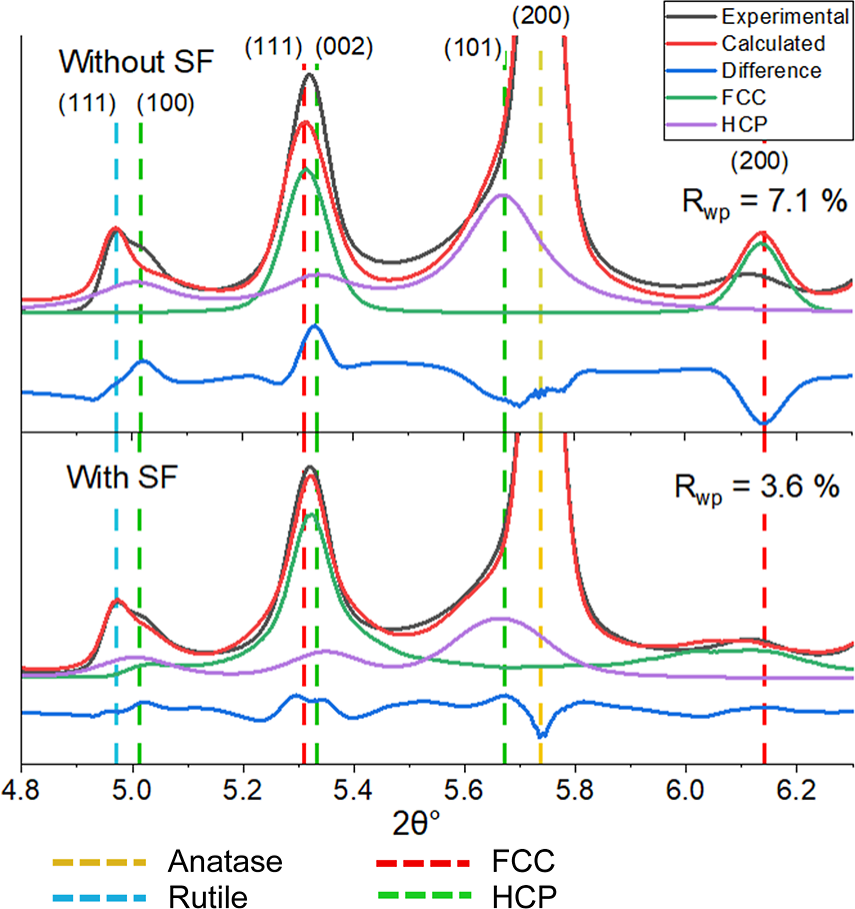
Rietveld refinement fit of the 3% Mn sample after reduction where the program struggled to fit the FCC (111) peak due to the absence of the (200) peak in the experimental data when modeling without stacking faults (SF). The inclusion of stacking fault models of the FCC/HCP phases resulted in an improved fit with reduced *R*_wp_. Measurements at 0.1896 Å.

The results of the refinements for the FCC/HCP Co stacking faulted phases are provided in Table 1, with further details on the support available in the appendix in Table S1. It was found that increasing the Mn loading from 0, to 3, to 5 wt % caused the wt % of the FCC phase to decrease, and that of the HCP phase to increase. Previous research highlights that the HCP phase is more active than the FCC phase, therefore this increasing quantity of HCP phase may explain the increase in CO conversion typically observed in Mn promoted catalysts.^9,18,49^ The extent of faulting, both intrinsic and extrinsic, decreased with Mn loading, indicating more ordered FCC/HCP structures.

**Table 1:** Lefthand Side Contains Quantitative Rietveld Refinement Results of the Samples After In Situ Reduction Where Stacking Fault Models were Implemented for the FCC and HCP Co Phases^a^

Mn		FCC Co with stacking faults			HCP Co with stacking faults		CoO		Co_3_O_4_	
Wt %	*R*_wp_(%)	Wt %	*i*	e	Wt %	*P*_c_	Wt %	LP (Å)	Wt %	LP (Å)
0	3.4	8.1	0.125	0.125	2.1	0.15	0.7	4.333	-	-
3	3.6	6.3	0.09	0.06	3.7	0.2	0.7	4.330	-	-
5	3.6	5.5	0.083	0.068	4.7	0.2	1.3	4.332	2.6	8.650
10	3.8	0.6	0.09	0.01	0.4	0.1	10.8	4.316	14.9	8.619

a Intrinsic and extrinsic faulting (*i* and *e*) decreased with increasing Mn loading in the faulted FCC phase. Righthand side contains results weight percentage and lattice parameters (LP) for the Co oxide phases. Increasing CoO is found with increasing Mn loading >5 wt %.

CoO content is observed to increase with greater Mn loading, particularly >5 wt %, since reduction was inhibited at these higher loadings under the conditions employed here. In the 10% Mn sample, almost all the Co content was present as CoO and Co_3_O_4_. The total summed Co content in the Co oxide phases for the 10% Mn sample was much higher than the 10 wt % Co known to be present, suggesting that Mn was incorporated in mixed oxide spinels (Co_*x*_Mn_3‑*x*_O_4_ and Co_*x*_Mn_1‑*x*_O). This correlates with previous research, where similar increases in lattice parameters were observed.^21^

### Stacking Fault Analysis in Structured Forms of the Catalyst Post Reaction

The occurrence and spatial distribution of stacking faults were studied on catalysts extracted after 300 h of reaction under FT conditions. XRD-CT measurements were performed on the catalysts preserved in their active state due to a coating of wax products that prevented oxidation.^18^ A comparison of the Rietveld refinement fit of the 0% Mn catalyst with and without simulated stacking fault models is presented in Figure 4. The inclusion of stacking faults resulted in an improved fit, enabling the fitting of the FCC (111) peak while accounting for the diminished FCC (200) peak. Furthermore, the faulted HCP phase was able to account for the intensity of the (100) peak at 3.61°, although it was not able to accurately replicate the broad (101) peak at 4.07°, which appears as a weak shoulder on the anatase (200) peak.

**Figure 4:**
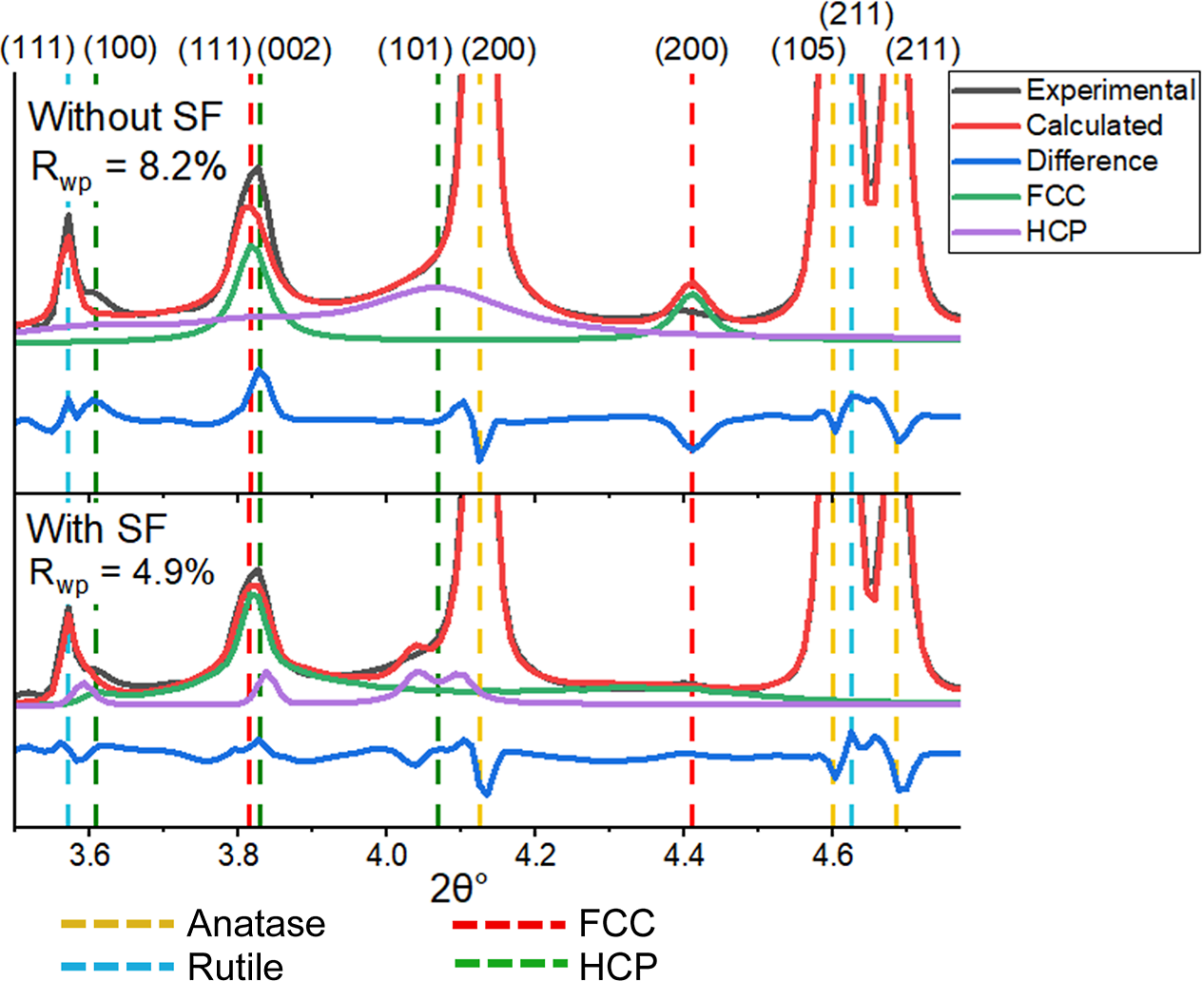
Rietveld refinement fit of the 0% Mn sample after 300 h reaction where inclusion of stacking faults resulted in an improved fit. Measurements at 0.1362 Å.

The results from the Rietveld refinement for the Co phases are also presented in Table 2, and details of the support are available in Table S2. Similar to the in situ reduction results, the probability of intrinsic faults in the FCC phase decreased with increasing Mn loading from 0 to 3% Mn, indicating increasing order. Increased Mn loading led to increased extrinsic faulting, where 45% of the stacking faults in the 0% Mn sample were extrinsic compared to 85% in the 3% Mn sample. This maxima in extrinsic faulting at 3% Mn coincided with the onset of Co_2_C formation, strongly suggesting that the extrinsically faulted surfaces facilitated the adsorption and activation of carbon-containing species. Alternatively, it is possible that the extrinsic faults were acting as nucleation sites that promoted Co_2_C nucleation. At ≥3% Mn, Co_2_C is the dominant phase, and its crystallite size increases slightly with increasing Mn loading (from 8.7 nm at 3% Mn to 10.0 nm at 10% Mn). Previous catalytic performance results demonstrate that, at this “tipping point” of 3% Mn, a drop in CO conversion occurs, back to the levels of the unpromoted catalyst (Figure S3).^18^ Increasing the Mn loading to 5 or 10% does not further affect the CO conversion. This loss of activity occurs concurrently with a change in product distribution, with a significant portion of C_5_^+^ selectivity replaced by olefins and alcohols.^18^

**Table 2:** Rietveld Refinement Results of the Samples after Reaction for 300 h Where Stacking Fault Models Were Implemented for the FCC and HCP Co Phases^a^

Mn		FCC with stacking faults			HCP with stacking faults		Co_2_C	
Wt %	*R*_wp_ (%)	Wt %	*i*	*e*	Wt %	*i*	Wt %	CS (nm)
0	4.9	5.9	0.11	0.09	1.7	0.2	-	-
1	5.4	6.3	0.11	0.09	1.4	0.2	-	-
2	5.4	6.1	0.0675	0.0825	1.6	0.2	-	-
3	5.7	2.2	0.0225	0.1275	2.1	0.1	4.6	8.7
5	6.1	-	-	-	0.3	0.0001	9.8	9.0
10	6.8	-	-	-	0.4	0.0001	9.1	10.0
10^a^	5.7	5.5	0.09	0.11	1.9	0.2	-	-

a Reduced at 450 °C during preparation. CS (crystallite size) of the Co_2_C phase is presented.

An additional 10% Mn sample is reported which was reduced at a higher temperature of 450 °C (compared to 300 °C). This sample, described as 10^a^ in Table 2, exhibited Co FCC/HCP faulting like the 0% Mn sample, and also yielded a similar catalytic performance to the 0% Mn sample (Figure 1). This can be explained by the large quantity of MnTiO_3_ observed in the XRD (26.4 wt %), likely formed during the initial high temperature reduction, and presumably inhibiting the promotional effects of Mn, more details of which can be found in the recent work published by Paterson et al.^50^ The refined parameters of the MnTiO_3_ phase are presented in Table S3. The 5 and 10% Mn samples did not contain FCC Co but contained a minimal amount of HCP Co (0.3–0.4 wt %). This was due to the Co being predominantly contained in the Co_2_C phase. The HCP phase had a faulting probability of almost zero (0.0001) in these samples, which could be due to the similar AB stacking of the HCP and Co_2_C phases.

Figure 5 illustrates the FCC and HCP Co wt % as a function of depth within the trilobe pellets. It was found that the FCC wt % increased toward the center of the 3% Mn sample. Furthermore, small amounts of FCC Co were only present at the center of the pellet for the 5 and 10% Mn samples. A higher proportion of HCP Co was consistently found at the center of the pellets, particularly in the 5 and 10% Mn samples, where only a very small amount of HCP was present in the periphery. Conversely, Co_2_C forms in preference on the periphery of these trilobe pellets, evidenced by our previous study.^18^ This could therefore be due to a higher H_2_ concentration at the center, due to the diffusion limitations of CO when compared to H_2_ into the 1.6 mm pellets which were observed previously and were also found to lead to CoO on the periphery.^51^ Little variation of FCC Co was found in the other samples with depth, and the differences are within error.

**Figure 5:**
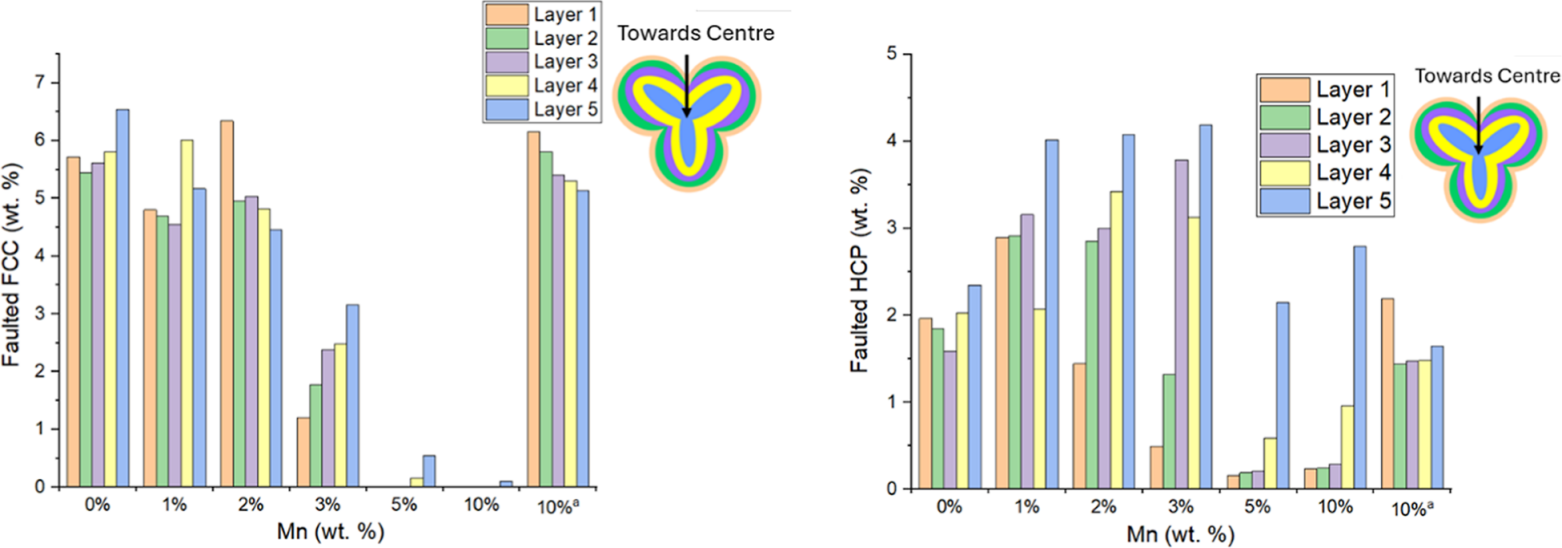
(Left) refined weight percentages of the FCC phase of the samples as a function of depth. The FCC wt % was relatively constant (5–7 wt %) for the 0–2% Mn sample but decreased for the 3–10% Mn samples. (Right) refined weight percentages of the HCP as a function of depth within the catalyst pellets. Higher HCP wt % was found at the center of the pellets.

Spatial maps of the optimized stacking fault probabilities are presented in Figure 6 for the different samples. Graphical representations of the maps are presented for FCC intrinsic and extrinsic faulting in Figure S3 and HCP intrinsic faulting in Figure S4. The presence of low amounts of FCC Co in the 5 and 10% samples prevented the mapping of the stacking fault probabilities. A reduction in intrinsic FCC stacking fault probability was observed with increasing Mn in the 0–3% Mn samples. Conversely, extrinsic faulting increased in the 3% Mn sample, particularly in the center, while intrinsic faulting continued to decrease. This increase in extrinsic faulting indicated that a higher proportion of HCP domains were present within the faulted FCC phase with increasing Mn loading. Moreover, a lower degree of faulting was observed at the center of the 3% Mn sample, and in its place highly ordered HCP Co was present and coincided with the initiation of Co_2_C formation.^18^ This was likely due to the similar ABAB stacking sequences.^9,14^ Furthermore, the probability of extrinsic faults in the HCP phase was higher at the center (0.05–0.15) with a refined weight percentage of approximately 2 wt %. The probability of extrinsic faults in the HCP phase on the periphery of the 5 and 10% Mn pellets were found to be close to zero as illustrated in Figure S4 but the HCP weight percentages were low (<0.5 wt % as found in Figure 5). These samples had little FCC/HCP Co content at the periphery due to the increased carbide content.

**Figure 6:**
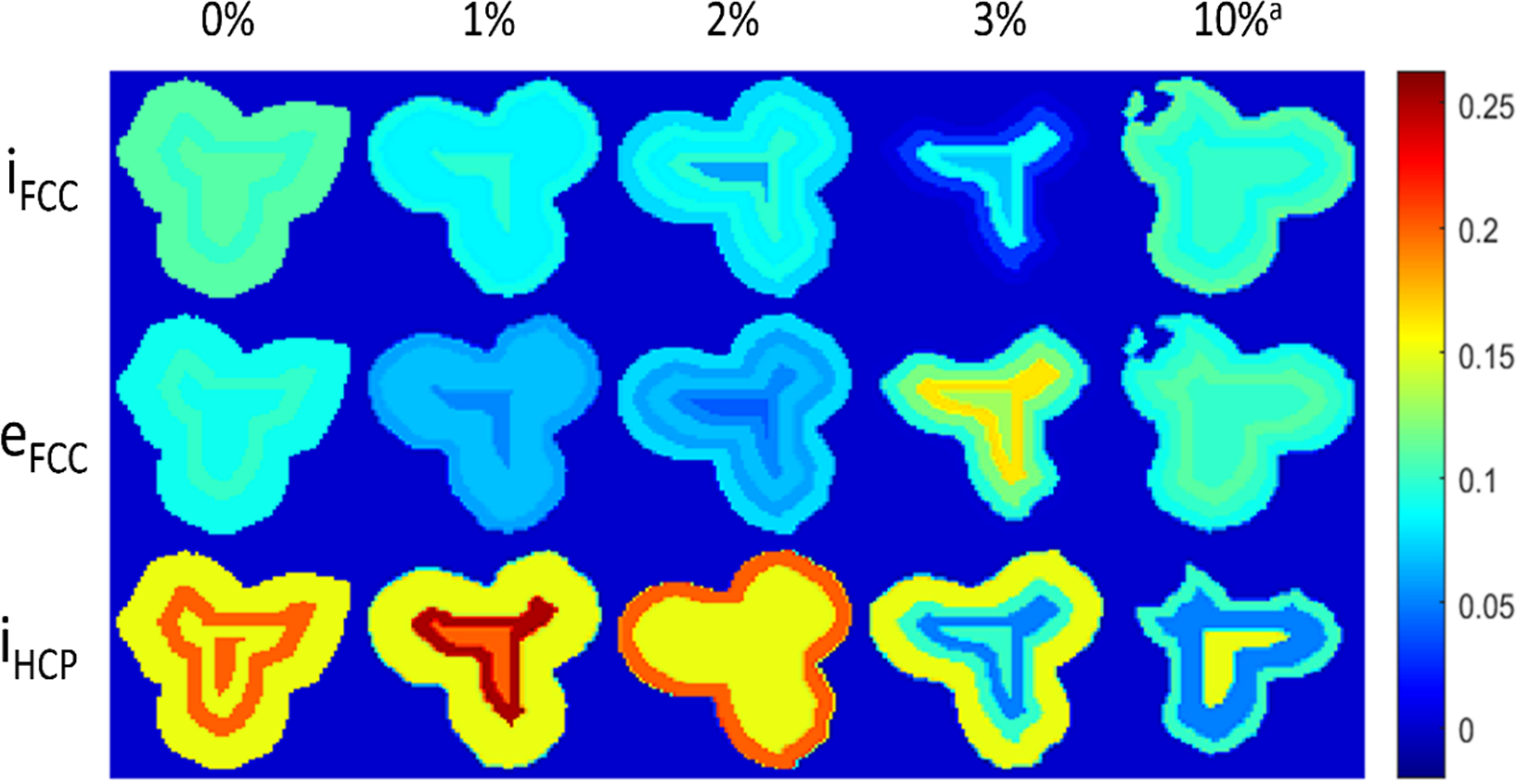
XRD-CT maps of the stacking fault probabilities of the FCC/HCP phases within the pellets. From 0 to 2% Mn, FCC intrinsic and extrinsic faulting decreased. However, the 3% Mn catalyst exhibited an increase in FCC extrinsic faulting and a decrease in HCP intrinsic faulting in the center.

## Summary and Conclusions

In order to better understand the correlation between structure and function in Co-based catalysts, analysis or modeling of the diffraction data has often been performed considering discrete FCC and HCP particles, but the presence of intergrown FCC/HCP species with stacking faults has been observed in the literature.^9,43^ Indeed, it is likely that such intergrown structures are the norm rather than the exception since some of the determined crystallite sizes of the polymorphs would likely be too small for them to be stable under operating conditions.^51,52^ However, as was also shown here, information on the crystallite size, is very difficult to extract reliably due to the difficulty in distinguishing between the two phases, given that reflections for the two phases overlap with each other and those of the support (particularly anatase). This makes it difficult to understand the effect that increased Mn loading has on Co polymorph formation. By modeling the Co FCC/HCP faulting via the creation of stacking sequences through supercells with varying degrees of faulting from ideal FCC and HCP sequences, following a similar approach to recent studies^47,48^ it was possible to greatly improve the fit of the experimental data (typically by 4–5% in terms of *R*_wp_), enabling a more accurate and representative understanding of the proportion of FCC and HCP domains and how this changes with [Mn]. However, it is important to note the profiling assumes an average distribution of the Co environments and does not differentiate between polydisperse crystallites with different degrees of faulting.

It was found from the PXRD data of the reduced powdered catalysts that the faulted FCC phase weight percentage decreased with increasing Mn loading while the HCP phase weight percentage increased, shown in Figure 7a. This in turn seems to correlate at low (≤2%) Mn loadings with improved CO conversion/C_5+_ yields, but at higher Mn loadings (>2%) with increasing amounts of Co_2_C, as in Figure 6b. This results in a selectivity change to alcohol and olefins, as previously observed for these samples.^18^ The stacking fault probability of the faulted FCC phase in the reduced samples also varied with increasing Mn loading from approximately 25% total faulting (intrinsic plus extrinsic) at 0% Mn to 15% at 3 and 5% Mn loading, shown in Figure 7a. On the other hand, the stacking fault probability in the faulted HCP phases was similar in proportion to the FCC phase but increased with increasing Mn loading, plateauing in the 3% Mn sample at a value of 20% (refer to Table 1). This was only slightly higher than the 15% faulting probability in the 0% Mn sample.

**Figure 7:**
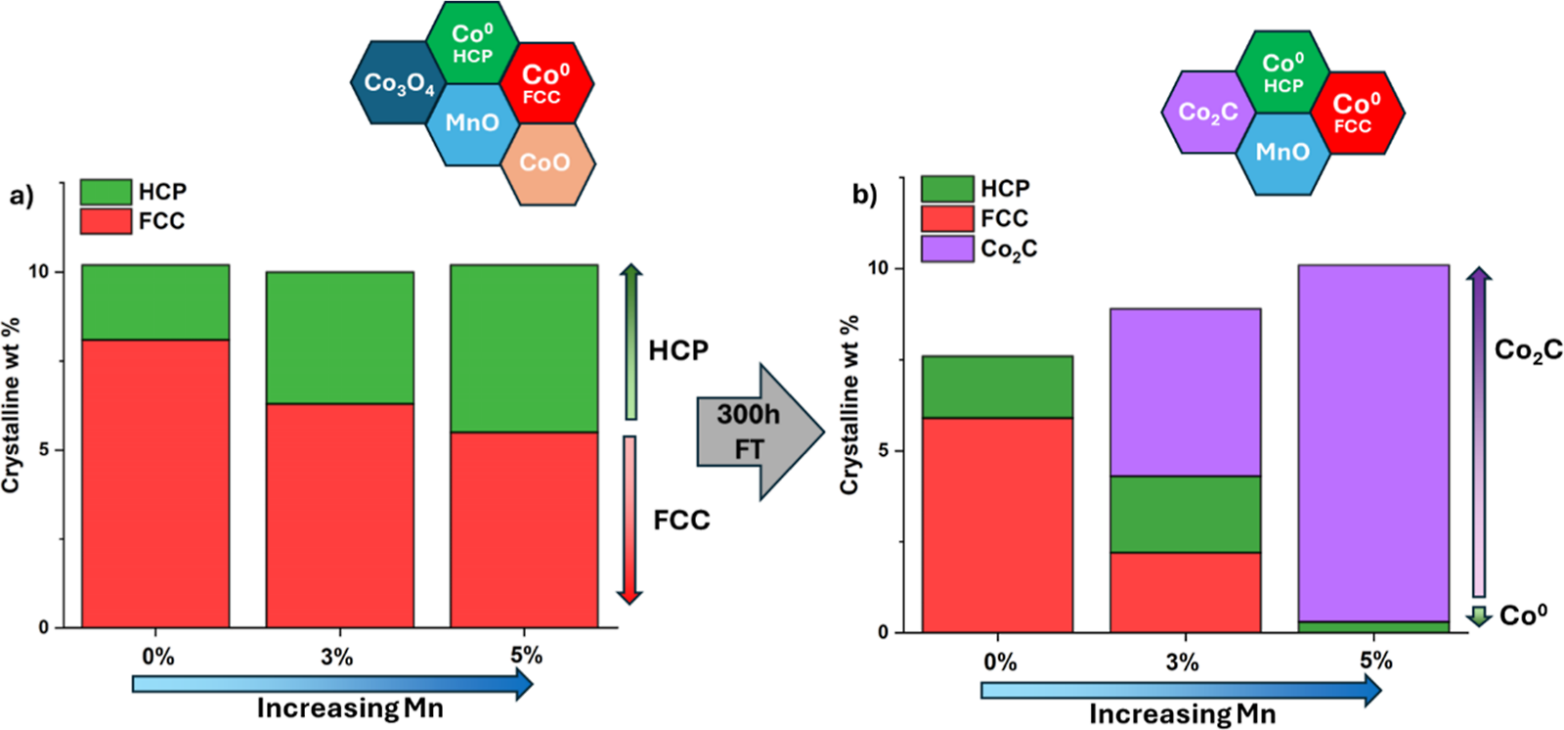
Refined crystalline weight percentages of the Co^0^ FCC and HCP phases, and the Co_2_C phase, as a function of Mn loading, in (a) the reduced catalysts and (b) the same catalysts after 300 h of reaction under Fischer-Tropsch (FT) conditions. A graphic highlights the Co and Mn phases present in each case.

It was found in the samples recovered and imaged postreaction that more HCP Co was present in the 3% Mn sample, this going on to form Co_2_C once the loading of Mn is equal to, or above, 3% (as in Figure 7b). Furthermore, extrinsic faulting in the FCC phase increased with Mn loading, indicating a greater proportion of HCP domains within the faulted FCC structure (Figure 8b). This indicates a phase transformation from extrinsically faulted FCC to HCP produced via reduction and possibly also during the early stages of reaction, followed by conversion to Co_2_C under operating conditions and with time. This transformation from HCP to Co_2_C is facilitated by the same AB cobalt layer stacking sequence in both phases. Moreover, the XRD-CT data revealed that a more ordered HCP phase was present at the center of the 3% Mn sample, which was the Mn loading at which significant Co_2_C formation is first observed. These data therefore suggest that extrinsic faults play a role in facilitating carbon adsorption and activation.

**Figure 8:**
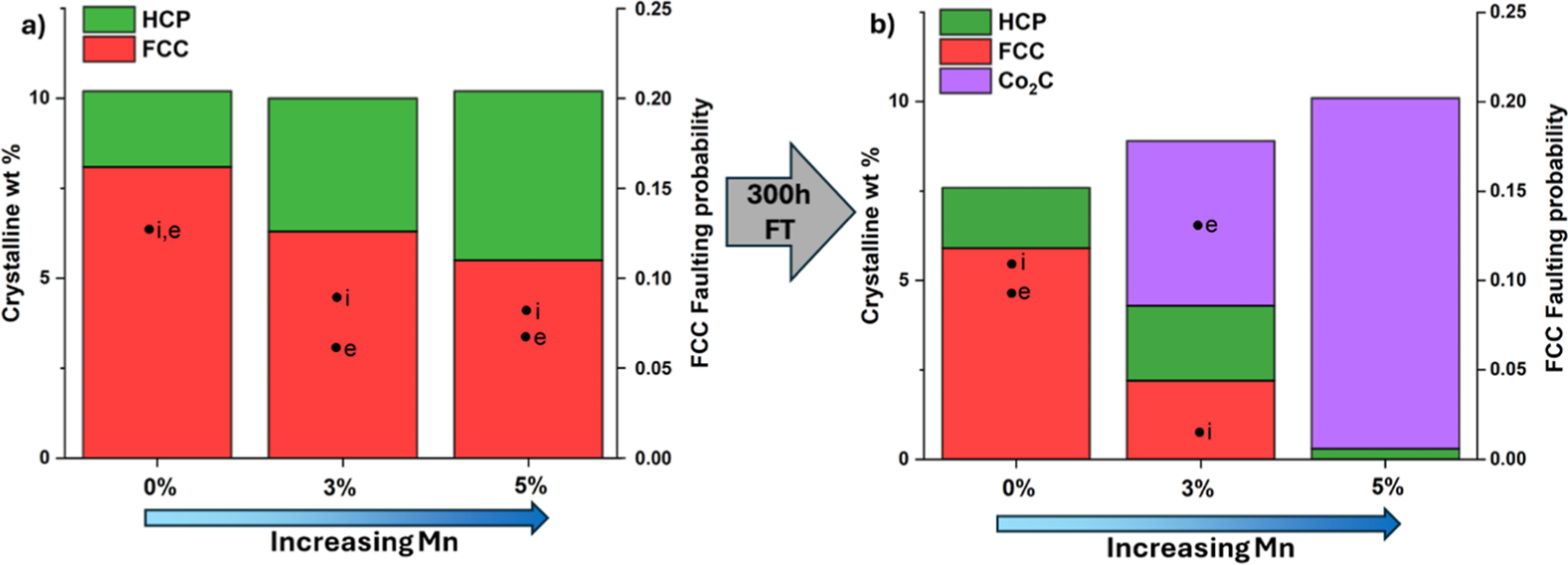
Refined crystalline weight percentages of the Co^0^ FCC and HCP phases, and the Co_2_C phase, as a function of Mn loading, in (a) the reduced catalysts and (b) the same catalysts after 300 h of reaction under Fischer-Tropsch (FT) conditions, with a secondary axis showing the calculated FCC faulting probability for intrinsic (i) and extrinsic (e) faulting for the FCC phase. These values can be summed to obtain the total faulting probability.

However, the modeling of stacking faults revealed the role of Mn in enhancing the weight percentage of HCP in samples with lower Mn loading, ultimately facilitating the formation of Co_2_C in samples with >3% Mn loading under reaction conditions. Notably, the 3% Mn sample demonstrated the highest selectivity for alcohol and olefin production, as reported previously with the data reproduced for ease of reference in Figure 1. This analysis establishes a clear correlation between increasing Mn content and the development of HCP domains, which contribute to Co_2_C formation and synergistically contribute to achieving maximum alcohol and olefin selectivity. Under FTS conditions, HCP Co has been shown to demonstrate greater C_5+_ selectivity, while DFT calculations indicate that Co_2_C facilitates nondissociative adsorption of CO, resulting in enhanced selectivity for oxygenates.^15,53^

Overall, this study reveals the importance of quantitatively analyzing Co stacking faults for a more accurate understanding of the presence and role of FCC/HCP Co^0^ domains in Co FT catalysts. More extrinsic faulting in the FCC Co phase and more HCP Co was detected with increasing Mn in reduced catalytic powders, which also aligned with the presence of Co_2_C in the reacted catalyst and increased alcohol and olefin selectivity. The growing formation of stacking faults may be attributed to the increasing extent of Co–Mn interactions and the resulting smaller Co particle sizes with higher Mn loading, as identified in a recent electron microscopy study.^54^ Previous studies have demonstrated that increasing Mn up to 2% improves CO conversion and C_5+_ yields.^55^ According to the findings in this work, this likely coincides with an increase in HCP Co domains, although it should be noted that the overall crystallite size also decreases.^15^ As such, it remains uncertain whether the increasing presence of the HCP phase directly contributes to the improved performance or if this is correlated with a reduction in FCC crystallite size.^56^ Further research is required to investigate the exact role of Mn on stacking fault occurrence and the specific effect of Co FCC/HCP intrinsic and extrinsic faults on activity and selectivity.

## Supplementary Material



## Notes

Further details regarding the building of models to create faulted structures and additional tables/figures containing bulk and spatially resolved phase composition data (PDF)
