# Supplementary material for: Mn-Promoted Co/TiO2 Catalysts: Quantitative Analysis of Cobalt Polymorphs and Stacking Faults and Its Effect on Fischer-Tropsch Synthesis Performance
Source: ACS Catal. 2026 Feb 5;16(4):3296–306. doi: 10.1021/acscatal.5c07197 (PMC12930387; doi:10.1021/acscatal.5c07197)
Supplement: Supplementary file 1 [file cs5c07197_si_001.pdf]

<u>**Supplementary Information**</u>

Mn-Promoted Co/TiO_2 Catalysts: Quantitative Analysis of Cobalt Polymorphs and Stacking Faults and its Effect on Fischer–Tropsch Synthesis Performance

Danial Farooq[1,2], Lucy Costley-Wood[1,2], Sebastian Stockenhuber[1,2], Antonis Vamvakeros[3], Stephen W. T. Price[3], Lisa J. Allen[1,2], Jakub Drnec[4], James Paterson[5], Mark Peacock[5], Daniel J. M. Irving[6], Philip A. Chater[6], Andrew M. Beale*[1,2,3]

[1]*Department of Chemistry, University College London, 20 Gordon Street, WC1H 0AJ, UK*

[2]*Research Complex at Harwell, Rutherford Appleton Laboratories, Harwell Science and Innovation Campus, Harwell, Didcot, OX11 0FA, UK*

[3]*Finden, Building R71, Harwell Campus, Oxfordshire, OX11 0QX, UK*

[4]*European Synchrotron Radiation Facility, ID 31 Beamline, BP 220, F-38043 Grenoble Cedex, France*

[5]*BP, Applied Sciences, Innovation & Engineering, Saltend, Hull, HU12 8DS, UK*

[6]*Diamond Light Source Ltd, Diamond House, Harwell Campus, Didcot, Oxfordshire, OX11 0DE, UK*

Email: Andrew.beale@ucl.ac.uk

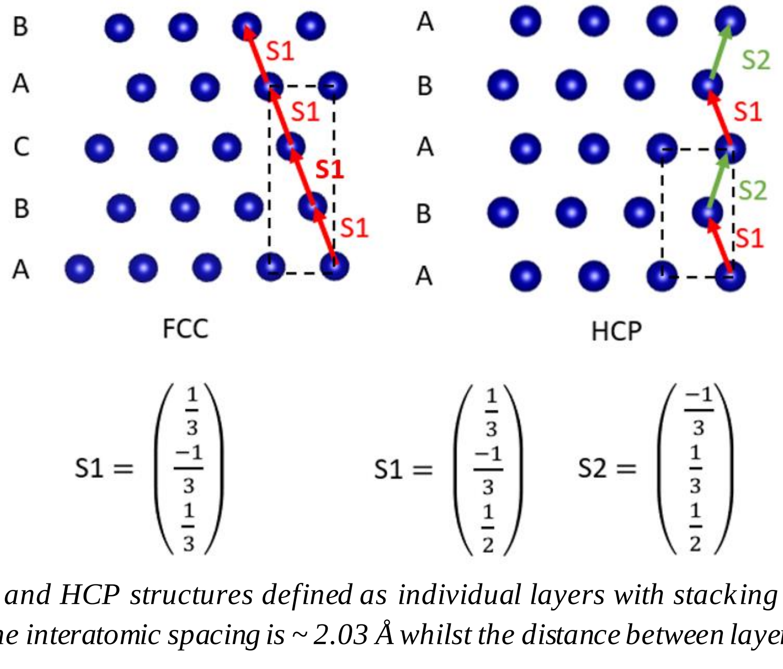

Figure S1. FCC and HCP structures defined as individual layers with stacking vectors defined between them. The interatomic spacing is ~ 2.03 Å whilst the distance between layers ~ 2.51 Å.

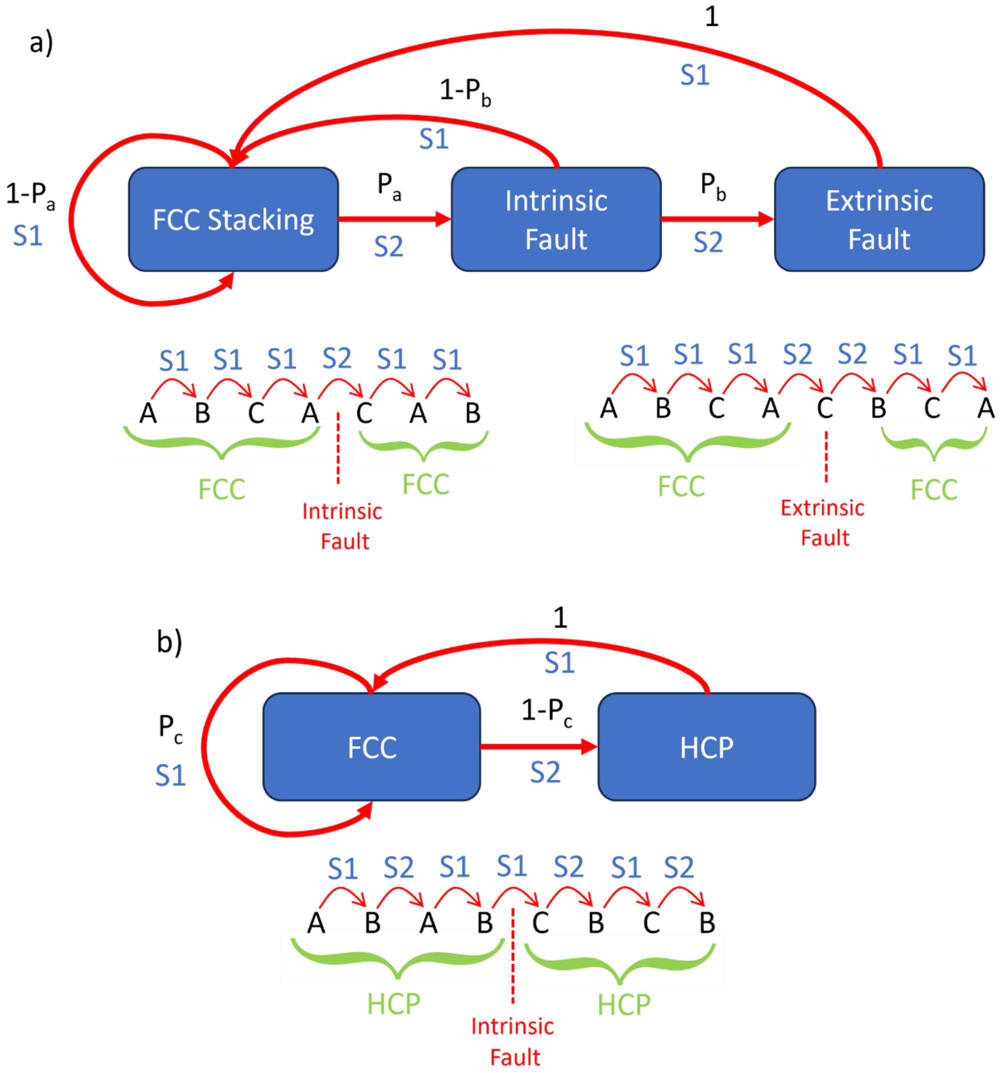

Figure S2. a) The model of the faulted FCC structure where P_a represents the probability of an intrinsic or extrinsic fault and P_b is the probability of that fault becoming an extrinsic fault. b) The model of the faulted HCP structure where P_c represents the probability of an intrinsic fault occurring.

$$i = P_a\,(1 - P_b\,) \tag{1.1}$$

$$e = P_a\,(P_b\,) = P_a - i \tag{1.2}$$

Table S1. XRD Refinement results of the support phases in the catalysts after in situ reduction collected at I15-1. There is little change in the support lattice parameters (LP (Å)), crystallite size (nm) and weight percentages. The 10 % Mn sample has a much lower TiO_2 wt. % due to the increase in Mn content in this sample.

	**Anatase**				**Rutile**			
**Wt%**	**Wt%**	**LPA (Å)**	**LPB (Å)**	**CS (nm)**	**Wt%**	**LPA (Å)**	**LPB (Å)**	**CS (nm)**
0	78.8	3.787	9.507	18.9	10.2	4.595	2.959	29.6
3	80.0	3.791	9.512	18.3	9.2	4.599	2.962	28.7
5	78.1	3.790	9.508	17.6	8.3	4.598	2.961	27.6
10	68.1	3.791	9.506	13.1	5.1	4.598	2.967	23.9

Table S2. XRD Refinement results of the support phases in the catalysts collected after 300 h of reaction. There is little change in the support lattice parameters (LP (Å)), crystallite size (CS (nm)) and weight percentages. The 10 %[a] Mn sample, reduced at 450 °C, had a much lower TiO_2 wt. % due to the presence of $MnTiO_3$.

**Mn**	**Anatase**				**Rutile**			
**Wt%**	**Wt%**	**LPA (Å)**	**LPB (Å)**	**CS (nm)**	**Wt%**	**LPA (Å)**	**LPB (Å)**	**CS (nm)**
0	79.2	3.785	9.503	22.2	13.2	4.593	2.959	47.9
1	80.1	3.786	9.505	21.8	12.3	4.594	2.959	44.3
2	81.4	3.785	9.502	20.9	10.9	4.593	2.959	43.1
3	81.0	3.785	9.503	20.5	10.7	4.593	2.959	43.9
5	80.3	3.785	9.502	20.1	10.0	4.593	2.959	42.6
10	83.1	3.786	9.500	14.6	7.94	4.593	2.959	29.9
10 [a]	63.9	3.786	9.502	16.4	2.3	4.596	2.958	36.2

Table S3. The refined parameters of the $MnTiO_3$ phase which was present only in the 10 % Mn sample (reduced at 450 °C). Weight percentages, lattice parameters (LP (Å)) and CS (crystallite size (nm)) are presented.

**Mn**	**$MnTiO_3$**				
**Wt. %**	**Wt. %**	**LPA (Å)**	**LPB (Å)**	**LPC (Å)**	**CS (nm)**
10	26.4	5.1	5.1	14.3	20.4

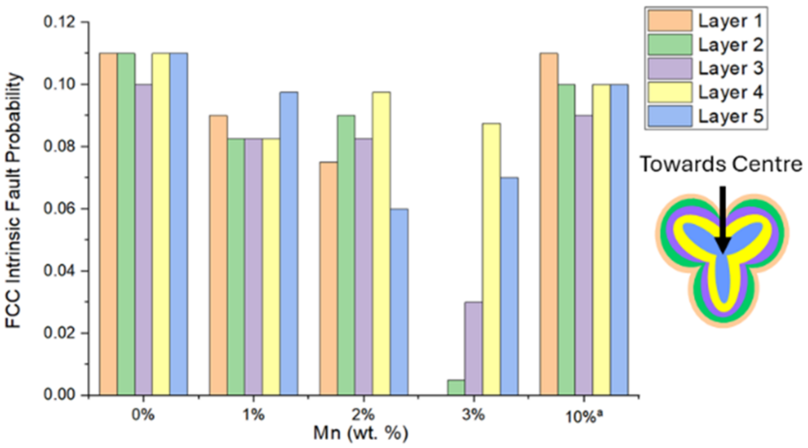

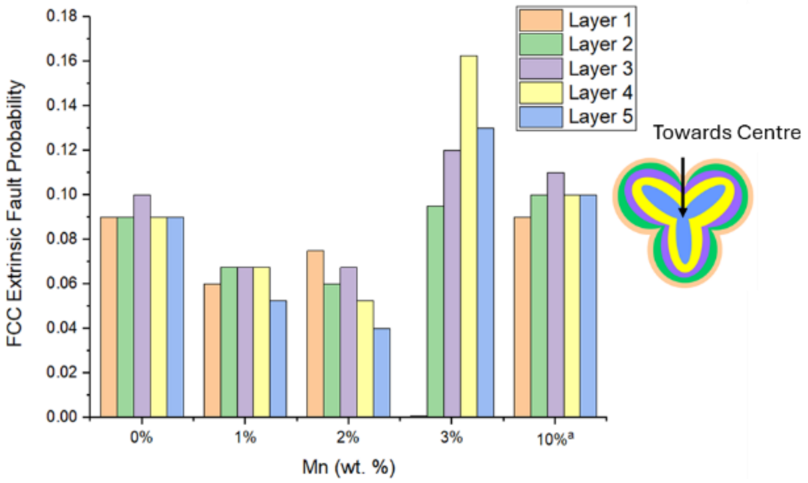

Figure S3. (Top) FCC intrinsic fault probability as a function of depth of the different samples. The FCC intrinsic faulting decreased with increasing Mn loading from 0 - 3 % Mn.(Bottom) FCC extrinsic fault probability as a function of depth. There is a decrease in faulting from 0 – 2 % Mn however faulting probability increases in the 3 % Mn samples which corresponds with increasing Co_2C wt. %.

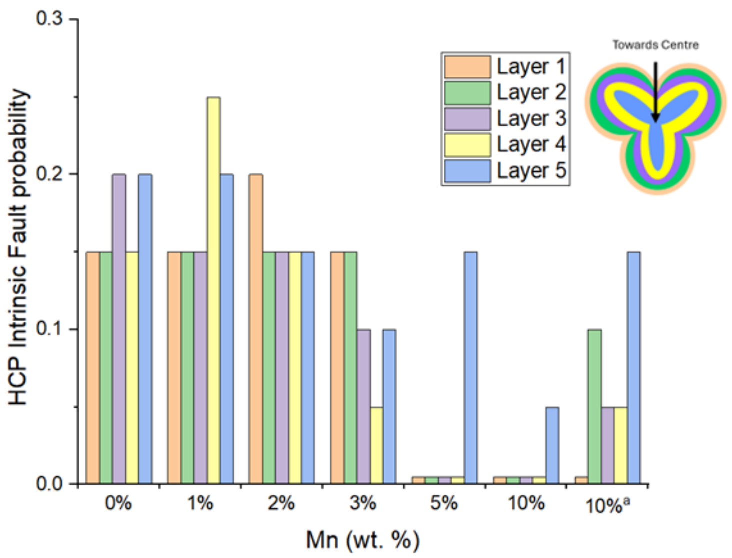

Figure S4. HCP intrinsic fault probability as a function of depth of the different samples. Faulting decreased in the 3 – 10 % Mn samples corresponding to increased Co_2C wt. %.
